# Case report: Reproductive organ preservation and subsequent pregnancy for an infertility patient with lynch syndrome-associated synchronous endometrial cancer and colon cancer after treatment with a PD-1 checkpoint inhibitor

**DOI:** 10.3389/fimmu.2022.1010490

**Published:** 2022-10-17

**Authors:** Di Cao, Yu Gao, Rong-xin Zhang, Fu-long Wang, Cong Li, Miao-qing Wu, Yi-fan Liu, Dan-dan Li, Gong Chen

**Affiliations:** ^1^ Sun Yat-sen University Cancer Center, State Key Laboratory of Oncology in South China, Collaborative Innovation Center for Cancer Medicine, Guangzhou, China; ^2^ Department of Colorectal Surgery, Sun Yat-sen University Cancer Center, Guangzhou, China; ^3^ Department of Obstetrics, The Sixth Affiliated Hospital of Sun Yat-sen University, Guangzhou, China; ^4^ Biotherapy Center, Sun Yat-sen University Cancer Center, Guangzhou, China

**Keywords:** PD-1 inhibitor, organ preservation, lynch syndrome, pregnancy, case report

## Abstract

Currently, immune checkpoint inhibitors (ICIs) are the mainstay of treatment for Lynch syndrome patients. However, the tumor regression features in radiology and pathology are inconsistent for patients who are treated with ICIs, which sometimes confuses surgical decision-making. Here, we report a case in which a 36-year-old patient suffering from infertility was diagnosed with Lynch syndrome-associated synchronous endometrial cancer and colon cancer, and persistently enlarged left iliac paravascular lymph nodes were detected after receiving sintilimab treatment, a programmed cell death 1 (PD-1) receptor inhibitor. Fortunately, when she was about to undergo hysterectomy and bilateral salpingo-oophorectomy, intraoperative pathology examination did not reveal any cancer cells in these lymph nodes, and therefore, her reproductive organs were preserved. Later, the patient successfully conceived and gave birth to a healthy male neonate with no immune-related adverse events (irAEs) during an 11-month follow-up. This case indicates that surgeons should carefully inspect the imaging characteristics after immunotherapy and that organ preservation is possible even for patients who fail to achieve complete clinical regression, which is especially important for female patients of childbearing age.

## Introduction

Lynch syndrome (LS), characterized by germline mutations of the DNA mismatch repair (MMR) system, is the most common inherited cancer syndrome (1). The global incidence of LS is approximately 1/279, and almost all LS-associated cancers present deficient MMR (dMMR) and high-level microsatellite instability (MSI-H). Colorectal cancer (CRC) and endometrial cancer (EC) are the two most common LS-associated cancers (2). There are approximately 6000 new LS-EC cases each year, accounting for 9% of all new EC cases worldwide (3, 4). Previously, the treatments for LS-EC and LS-CRC were the same as those of general EC and CRC. Surgery is the first choice if the tumor is resectable. For unresectable CRC or EC patients, receiving translational therapies is necessary, including chemotherapy, radiotherapy, targeted therapy and immunotherapy, which provides opportunities for radical resection. Understandably, surgery for LS-EC patients permanently destroys fertility. This situation hit a turning point when Le et al. demonstrated that dMMR patients were particularly sensitive to pembrolizumab, a representative ICI drug, in 2015 (5). Thus, the 2018 National Comprehensive Cancer Network (NCCN) guidelines recommended that pembrolizumab should be used in patients with MSI-H or dMMR diseases, greatly benefiting LS patients during immunotherapy.

However, the different tumor regression features in radiology and pathology of patients treated with ICIs have influenced clinical decision-making for surgeons. For female patients, reproductive organ preservation is an inevitable issue. If clinical complete regression (cCR) is not achieved according to preoperative images, patients are obliged to undergo radical resection, which leads to permanent infertility. However, if pathological complete regression (pCR) is achieved, radical surgery should be avoided.

Additionally, PD-1 inhibitors (IgG4 antibodies) may influence fetal development during pregnancy by crossing the human placenta. Using anti-PD-1 antibodies in mice weakens the immune tolerance mediated by regulatory T cells (Tregs) and significantly increases miscarriage rates (6). This finding revealed a key role of the PD-1/PD-L1 pathway in maintaining maternal-fetal immune tolerance. Thus, PD-1 antibody is classified as an FDA pregnancy Category D drug (7).

In general, this case suggests that surgeons should focus on reproductive organ preservation in female patients receiving ICI treatment rather than only considering preoperative images. In addition, our case provides additional experience in using PD-1 antibody in patients who plan to conceive. This study was reported in agreement with the principles of the CARE Guidelines (8) and includes a reporting checklist as **Supplementary Material** (**Table S1**) (9).

## Case description

**Figure 1:**
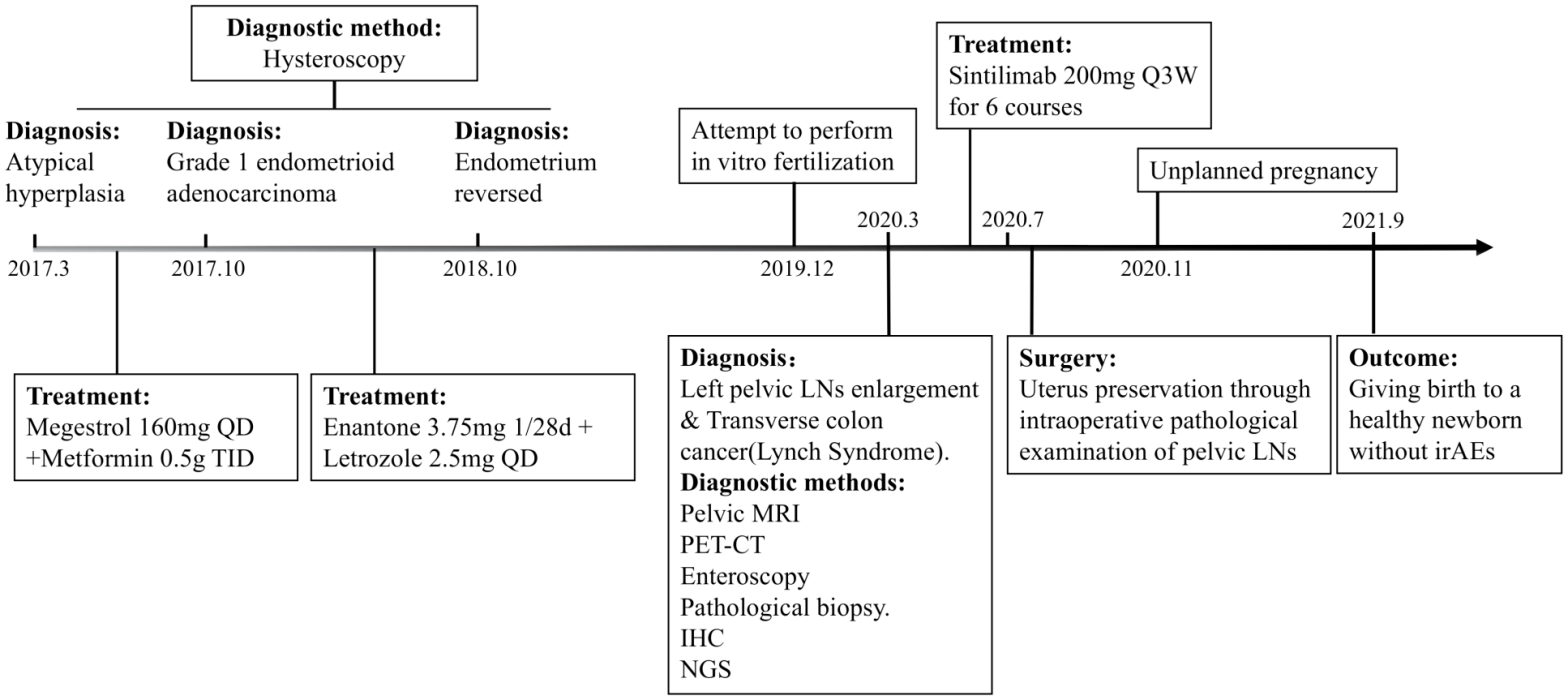
Timeline overview.

The patient was a 36-year-old woman who suffered from LS-EC and LS-CRC. She conceived naturally and successfully bore a male neonate after treatment with sintilimab, a PD-1 inhibitor. The timeline is shown in **Figure 1**. At the age of 32, she went to Fudan University Obstetrics and Gynecology Hospital because she had failed to conceive naturally for more than one year. She underwent hysteroscopy and was found to have atypical hyperplasia of the endometrium, so she was prescribed an initial regimen of megestrol 160 mg QD in combination with metformin 0.5 g TID. Seven months later, she underwent a second hysteroscopy, which detected that the endometrium had locally progressed to grade 1 endometrioid adenocarcinoma. Due to her strong desire to conceive, the patient refused the medical advice of hysterectomy. She was referred to Peking Union Medical College Hospital, and her medication regimen was changed to enantone 3.75 mg 1/28D + letrozole 2.5 mg QD. One year later, her endometrium had reversed, and she was advised to undergo *in vitro* fertilization (IVF). However, she did not adopt this suggestion for personal reasons, and over the following year, she still failed to conceive naturally without any contraception. In December 2019, she requested IVF at Xiangya Hospital of Central South University. Preoperative hysteroscopy did not show any endometrial abnormality, but she underwent additional pelvic magnetic resonance imaging (MRI) owing to her history of endometrial cancer, which showed that the left pelvic lymph nodes (LNs) were enlarged and considered metastases. In addition, her positron emission tomography-computed tomography (PET-CT) scan showed a hypermetabolic lesion in the transverse colon, which was later confirmed as a transverse colon (TC) adenocarcinoma by colonoscopy biopsy.

The patient came to our hospital in March 2020. We reexamined the colonoscopy results and identified the TC mass as a moderately to poorly differentiated adenocarcinoma. Of note, her immunohistochemistry (IHC) examination indicated the loss of MSH2 protein in both her EC and CRC lesions. Moreover, her father, aunt, and cousin all died of malignant tumors. Therefore, LS was highly suspected, and we conducted genetic testing, which revealed that both the EC and TC lesions were MSI-H and had somatic mutations of MSH2. More importantly, the patient also carried germline MSH2 mutations. The tumor mutation burden (TMB) of the EC lesion was 29.9 muts/Mb, and the TMB of the TC lesion was 77 muts/Mb. Based on her medical history and test results, the patient was diagnosed with LS.

**Figure 2:**
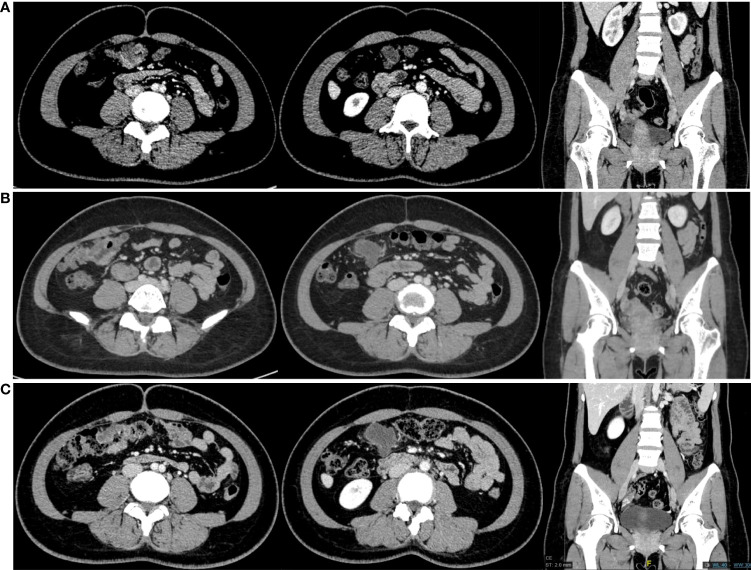
CT scans obtained from the patient. From left to right, each group of images shows the transverse colon lesion, para-transverse colon lymph nodes and the lymph nodes next to the left iliac vessels. **(A)** CT scans in March 2020. **(B)** CT scans in May 2020. **(C)** CT scans in July 2020.

Thoracoabdominal and pelvic CT were performed on March 16, 2020 (**Figure 2A**). CT images showed that the long diameter of the TC lesion was 32 mm, and there were enlarged LNs adjacent to the primary colon and left iliac vessels. The short diameters of the largest pelvic and pericolic LNs, which were considered metastatic LNs, were 12 mm and 18 mm, respectively. According to the results, we initiated neoadjuvant immunotherapy with sintilimab at a fixed dose of 200 mg every 3 weeks. From March to July 2020, the patient received 6 courses of immunotherapy. No significant immune-related adverse events (irAEs) were observed during the treatment. After the third course on May 5, 2020, the patient underwent enteroscopy and abdominal CT (**Figure 2B**). Both assessments suggested that the size of the primary and pericolic LNs had increased to 41 mm and 21 mm, respectively, compared with baseline, while the largest left pelvic LN had decreased to 8 mm. Because of our therapeutic goal of controlling the pelvic metastatic LNs, we decided to continue her neoadjuvant therapy. At the end of the sixth course on July 14, 2020, another abdominal CT was performed (**Figure 2C**) and showed that the TC lesion and pelvic LNs were stable, while the pericolic LNs had increased to 26 mm. In addition, her CEA serum level increased from 7.02 ng/ml to 19.17 ng/ml. At this time, we organized a multidisciplinary team (MDT) to discuss the case. According to the images, the enlarged left pelvic LNs most likely originated from EC metastasis. Although the TC lesion was confirmed as iCPD by the irRECIST method, it could reach R0 resection. Therefore, we decided to perform surgery. The surgical plan included radical TC resection, hysterectomy, bilateral salpingo-oophorectomy resection, pelvic LN dissection and retroperitoneal LN dissection.

On July 31, 2020, the patient underwent laparoscopic right hemicolectomy with radical lymphadenectomy. We did not observe any other lesions in the abdominal-pelvic cavity during intraoperative exploration. The tumor resided in the TC near the hepatic flexure and was approximately 6*5 cm in size. It possessed a tough texture and had invaded the serosa. The boundary between the tumor and the mesenteric LNs was unclear. There were several enlarged lymph nodes that extended from the left iliac vasculature to the left obturator area. Therefore, we performed an intraoperative gynecological consultation, and the gynecologist proposed total hysterectomy, bilateral salpingo-oophorectomy, and left pelvic lymphadenectomy. However, considering the patient’s desire to conceive and the possible effects of immunotherapy, we decided to send the left pelvic LNs for pathological examination before resection, and none of the 7 submitted pelvic LNs contained cancer cells. Subsequently, we communicated with her family about the possibility of pathological complete regression (pCR) and the risk of EC recurrence without hysterectomy. Ultimately, her family chose to preserve her uterus and bilateral adnexa and signed an informed consent document for the fertility preservation strategy. Postoperative pathological examination confirmed that the TC lesion was a poorly differentiated adenocarcinoma that had invaded through the muscularis propria into the pericolic tissues (pT3). Nerve tract invasion and intravascular cancer embolus were not observed. The resection margin was negative, and the tumor regression grade (TRG) was 2. No cancer tissue was observed in a total of 35 dissected LNs, including 28 pericolic LNs and 7 left pelvic LNs. The tumor deposits were also not detected.

After the surgery, the patient received adjuvant immunotherapy with a PD-1 inhibitor for 1 year. Although we told her to use contraception, she did not follow this advice because she had lost faith in natural conception. However, in December 2020, the patient unexpectedly found herself at 38 days’ gestation. Even though we informed the patient and her family of the possible adverse influences on the fetus, they still planned to continue with the pregnancy and complied with the obstetrician’s advice. There were no tumor-related conditions or gestational abnormalities, and in the 36^th^ week of gestation, the patient successfully gave birth to a male neonate by cesarean section. The Apgar score of the male neonate was 9, and his serum levels of G6PD, TSH, 17-OHP and PHE were normal. There were no irAEs in either the mother or the neonate during an 11-month follow-up, and the patient is still being followed closely.

## Discussion

We report the first case in which a female LS patient suffering from both EC and CRC successfully conceived and delivered after receiving treatment with sintilimab, a PD-1 antibody. Our report mainly highlights the complexity of reproductive organ preservation for LS-EC patients. After PD-1 antibody treatment, uterine preservation is still feasible even in EC patients with pelvic LN metastases, which diverges from the previous therapeutic principle that hysterectomy is usually unavoidable for these patients.

In recent years, infertility treatment strategies have become established and various in number. Nontumorous female patients can undergo ovarian stimulation, oocyte vitrification, and IVF to treat infertility. However, these practices are still limited for patients suffering from EC, which is the most frequent gynecological malignancy and severely influences young patients without a childbearing history (10, 11). The early detection of EC is extremely important for the reproductive organ preservation of patients. Diagnostic tools for early-stage EC detection are emerging, such as relative telomere length in cell-free DNA, glandular cells detected at preoperative cervical smear, and transvaginal ultrasound (12–14). The treatment of early-stage EC is conservative and follows the conventional regimen of medroxyprogesterone acetate (MPA) and megestrol acetate (MA) (15). Previously, type I EC at the early stage was identified as the only histological type that can be addressed with a fertility-sparing approach. Nevertheless, with a deeper understanding of molecular oncology, the importance of molecular classification for EC treatment was gradually realized, and MSI was determined to be a fair prognostic factor for fertility-sparing treatment (16, 17).

Notably, LS-EC patients featuring MSI could benefit from PD-1 antibodies, and clinicians should take into account the possibility of CR, which means that non-early-stage young patients still have a chance to preserve their reproductive organs. It is worth noting that the pathological remission rate often exceeds the radiological remission rate in patients receiving PD-1 antibody treatment. For example, 13 patients with dMMR CRC achieved pCR after pembrolizumab treatment, but among them, only 1 patient achieved cCR (18). Furthermore, some patients may develop lesion enlargement during treatment, probably caused by T-cell infiltration into the tumor stroma. These studies suggest that imaging is insufficient as an absolutely accurate standard for evaluating PD-1 efficacy. Intraoperative pathological biopsy may provide additional information for better treatment decisions.

How PD-1 antibodies influence pregnancy has always been a topic of interest. Recently, several cases have reported successful pregnancy outcomes in patients treated with PD-1 antibodies in different cancer types, including metastatic melanoma, relapsed Hodgkin’s lymphoma and placental site trophoblastic tumors (6, 7, 19–27). Nevertheless, irAEs, such as intrauterine growth restriction, HELLP syndrome, placental insufficiency and low fetal heart rate, have occurred in some of these patients (28). Thus, whether conception is safe for patients after treatment with PD-1 antibody is still controversial. Current studies believe that blocking the PD-1/PD-L1 pathway will interfere with normal pregnancy because of its key role in maintaining maternal immune tolerance to the fetus, which is regarded as an allogeneic component. Fetal antigens can be processed and presented in the maternal body, leading to the activation and expansion of anti-fetus T cells (29). At the early stage of pregnancy, increased PD-L1 expression in trophoblast cells, decidual macrophages and decidual stromal cells on the maternal-fetal interface can inhibit the activity of T helper cells and the production of proinflammatory cytokines and facilitate the function of Treg cells, which mediate maternal-fetal immune tolerance. In mice, blockade of the PD-1/PD-L1 pathway inhibited Treg cell function and reduced the embryonic survival rate. In crab-eating monkeys, using high doses of nivolumab antibodies (10 mg/kg or more) resulted in significantly higher risks of fetal growth restriction and premature delivery.

However, the effect of PD-1 antibody on pregnancy is determined by multiple factors and needs comprehensive consideration. First, the administration doses and frequencies of PD-1 antibodies in clinical settings are much lower and less than those in experimental settings. Even when exposed to high doses of PD-1 antibody, animals did not present increasing risks of fetal malformations, immunodeficiencies or neurological complications (30). Second, the effect of PD-1 antibody on the fetus is subject to the interval between the last dose of the antibody and the start of pregnancy. PD-1 antibody, as an IgG antibody, requires active transport to enter the placenta where the neonatal Fc receptor (FcRn) resides. FcRn on syncytiotrophoblast cells can bind to IgG antibodies and allow them to pass through the placental barrier. However, FcRn is rarely detected during the first 14 weeks of pregnancy, which restricts the transfer of IgG antibody (31). Thus, the timing of the last administration and the pharmacokinetics of the PD-1 antibody are also involved in its effect on pregnancy. Finally, the patient’s fertility intention and physical condition need to be taken into full consideration. Currently, the National Comprehensive Cancer Network guidelines recommend that all patients of reproductive age should use effective contraception during immunotherapy and maintain its use for at least 5 months after the last course of immunotherapy, which lacks adequate evidence and needs further exploration.

In conclusion, our report indicates that for young female patients with LS-EC, surgeons should be aware of the possibility of reproductive organ preservation. Intraoperative pathological biopsy is more conducive to guiding surgical decision-making for these patients. In addition, we recommend that patients should use contraception for a sufficient period after receiving PD-1 antibody treatments to reduce the incidence of irAEs.

## Data availability statement

The original contributions presented in the study are included in the article/**Supplementary Material**. Further inquiries can be directed to the corresponding authors.

## Ethics statement

Written informed consent was obtained from the individual(s) for the publication of any potentially identifiable images or data included in this article.

## Author contributions

DC collected the data and wrote the manuscript. DC, GY, CL, F-lW, R-xZ, D-dL, and GC was involved in the diagnosis and treatment of the disease. GY performed the histological obstetrical examination. D-dL and GC performed conception and design. GY, F-lW, R-xZ, CL, M-qW, Y-fL, D-dL, and GC reviewed and revised the manuscript. D-dL and GC supervised the review and approved the final version of the manuscript. All authors contributed to the article and approved the submitted version.

## Acknowledgments

We appreciated the gynecologist, Ting Wan and the department of pathology for consultations during treatment.

## Conflict of interest

The authors declare that the research was conducted in the absence of any commercial or financial relationships that could be construed as a potential conflict of interest.

## Publisher’s note

All claims expressed in this article are solely those of the authors and do not necessarily represent those of their affiliated organizations, or those of the publisher, the editors and the reviewers. Any product that may be evaluated in this article, or claim that may be made by its manufacturer, is not guaranteed or endorsed by the publisher.
