# Supplementary figures and images for: Case report: Reproductive organ preservation and subsequent pregnancy for an infertility patient with lynch syndrome-associated synchronous endometrial cancer and colon cancer after treatment with a PD-1 checkpoint inhibitor
Source: Front Immunol. 2022 Oct 17;13:1010490. doi: 10.3389/fimmu.2022.1010490 (PMC9618861; doi:10.3389/fimmu.2022.1010490)

Supplementary Material

# Supplementary Table 1

Table S1. The CARE checklist.


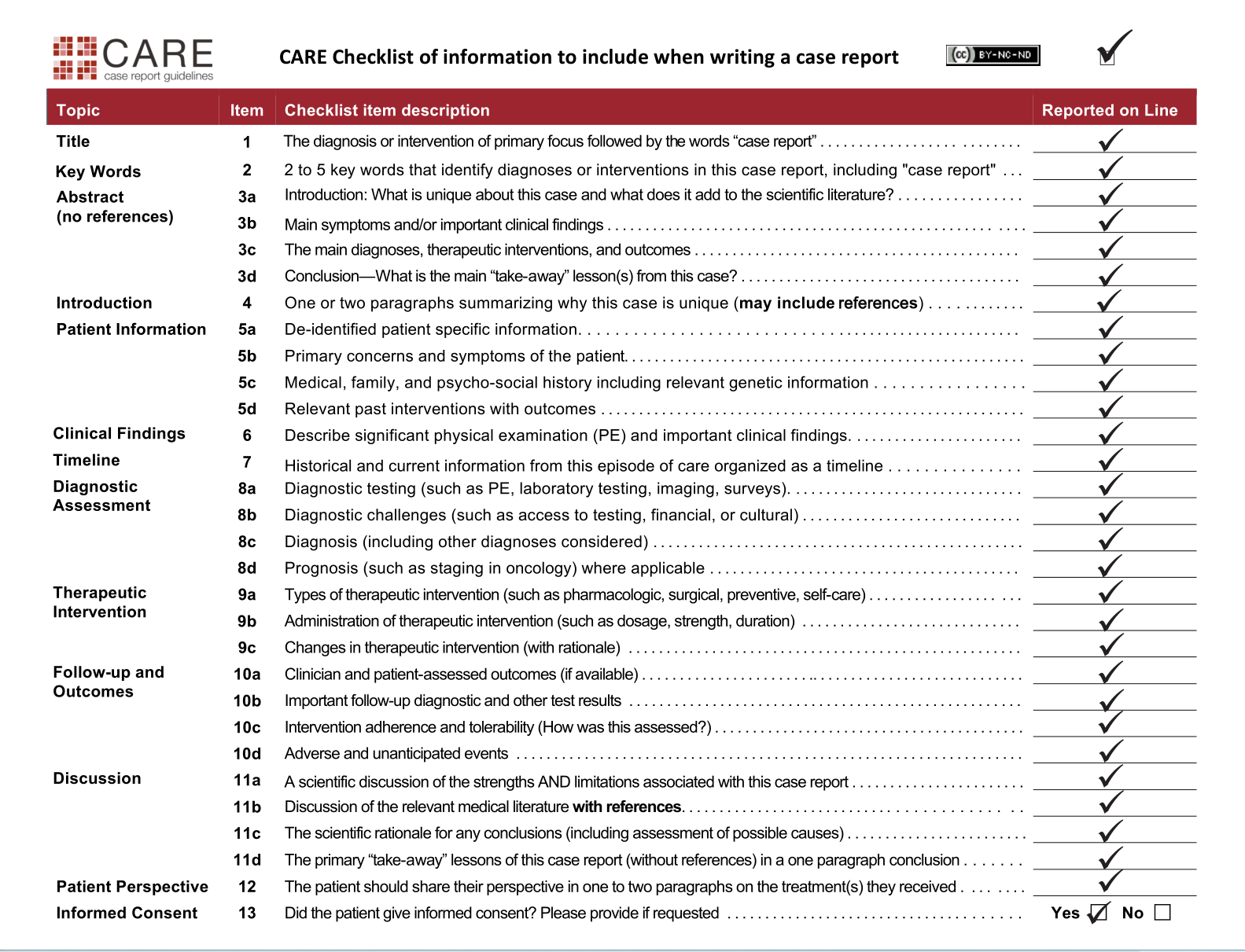

Supplement: Supplementary file 1 [file Table_1.docx]
